# Determination of the Neutron Lifetime Using Magnetically Trapped Neutrons

**DOI:** 10.6028/jres.110.050

**Published:** 2005-08-01

**Authors:** S. N. Dzhosyuk, A. Copete, J. M. Doyle, L. Yang, K. J. Coakley, R. Golub, E. Korobkina, T. Kreft, S. K. Lamoreaux, A. K. Thompson, G. L. Yang, P. R. Huffman

**Affiliations:** Harvard University, Cambridge, MA 02138, USA; National Institute of Standards and Technology, Boulder, CO 80303, USA; North Carolina State University, Raleigh, NC 27695, USA; Hahn-Meitner-Institut, Berlin, Germany; Hahn-Meitner-Institut, Berlin, Germany; Tulane University, New Orleans, LA 70118, USA; Los Alamos National Laborataory, Los Alamos, NM 87545, USA; National Institute of Standards and Technology, Gaithersburg, MD 20899, USA; North Carolina State University, Raleigh, NC 27695, USA; National Institute of Standards and Technology, Gaithersburg, MD 20899, USA

**Keywords:** magnetic trapping, neutron lifetime, superthermal neutron production, ultracold neutrons

## Abstract

We report progress on an experiment to measure the neutron lifetime using magnetically trapped neutrons. Neutrons are loaded into a 1.1 T deep superconducting Ioffe-type trap by scattering 0.89 nm neutrons in isotopically pure superfluid ^4^He. Neutron decays are detected in real time using the scintillation light produced in the helium by the beta-decay electrons. The measured trap lifetime at a helium temperature of 300 mK and with no ameliorative magnetic ramping is substantially shorter than the free neutron lifetime. This is attributed to the presence of neutrons with energies higher than the magnetic potential of the trap. Magnetic field ramping is implemented to eliminate these neutrons, resulting in an $833_{-63}^{+74}\;s$ trap lifetime, consistent with the currently accepted value of the free neutron lifetime.

## 1. Introduction

We present a progress report on an experimental program to improve the measurement of the neutron lifetime, *τ*_n_, using a technique with completely different systematic effects than previous measurements [1]. Ultracold neutrons (UCN) are produced by inelastic scattering of cold (0.89 nm) neutrons in a reservoir of superfluid ^4^He (the “superthermal” process). These neutrons are then confined by a three-dimensional magnetic trap. As the trapped neutrons beta decay, the resulting energetic electrons generate scintillations in the liquid He. Each decay is detectable with high effciency. Thus, *τ*_n_ can be directly determined by measuring the scintillation rate as a function of time.

For detailed information on the experiment, the reader is directed to the graduate thesis of S. N. Dzhosyuk [2] and Refs. [3] and [4]. This paper summarizes some highlights of the neutron trapping/lifetime data collected at the National nstitute of Standards and Technology (NIST) from the fall of 2002 until the summer of 2003.

## 2. Experimental Procedure

In order to correct our data for both time-dependent and time-independent backgrounds, data is collected in what we refer to as “trapping” and “non-trapping” runs. In a trapping run, the magnet is energized while neutrons are loaded into the trap. After the beam has been turned off, the neutron decay events are recorded. In non-trapping (or background) runs, the magnet is deenergized while the beam is on, then raised to the full value as the neutron beam is turned off. In this non-trapping case, the background events arising from neutron activation, neutron-induced luminescence, etc. should be the same. A difference in the count rate versus time between trapping (trapped UCN + backgrounds) and non-trapping (backgrounds only) runs should arise solely due to magnetically trapped UCN. If for some reason the backgrounds are not identical in the trapping and non-trapping runs, then the subtraction process will leave a residual difference that could mimic a trapping signal. Measurements made with natural abundance helium are used to conclusively determine that a putative trapping signal is, in fact, due to trapped neutrons.

Both trapping and non-trapping data was collected in a number of configurations that will be described below. Analysis of each data set is performed by integrating the pulse area of each digitized photomultiplier tube (PMT) signal and applying appropriate lower level threshold cuts on the area of the pulses. Since neutron-induced luminescence (occurring as single uncorrelated photons) is known to be present in the coincidence data with thresholds at single photoelectron levels, thresholds are set to require an area in each pulse equivalent to at least three photoelectrons.

**Fig. 1:**
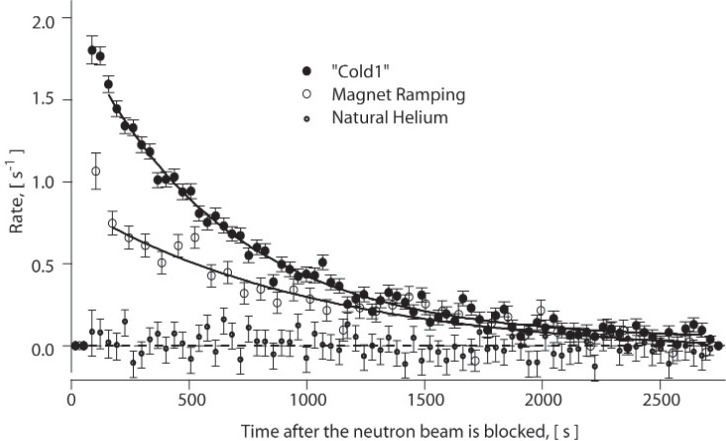
The difference of the trapping and non-trapping data collection runs taken at a helium bath temperature of *T* = 300 mK for approximately eight weeks of data (upper curve). The lower curve represents approximately 2 weeks of data taken with natural helium in the trapping region where one would expect no trapped neutrons due to neutron capture on ^3^He. The middle curve represents neutron lifetime data after removal of the marginally trapped neutrons. Details on each set of data and the corresponding fit parameters are described in the text. Each data set shown has the light directed into two PMTs with a threshold limit corresponding to at least three photoelectrons in each PMT.

A representative set of data (from approximately 8 weeks of data collection) is shown as the upper curve in Fig. 1. The curve is obtained by taking the difference between the trapping and non-trapping runs and then fit to the function *y* = *y*_0_ + *A* exp(− *t/τ*) with parameter estimates *y*_0_ = 0.04 ± 0.01 s^−1^, *A* = (1.94 ± 0.03) s^−1^ and $\tau =(621_{-17}^{+18})\;s$ with *χ*^2^ = 0.96, where *χ*^2^ is the reduced chi-squared value. As one can see, the lifetime obtained from this data is substantially shorter than the presently accepted value of the neutron lifetime (885.7 ± 0.8) s (1 *σ* uncertainty) [1]. Subsequent runs have led us to identify this systematic effect in our system as arising from marginally trapped neutrons.

## 3. Study of Systematics

A wide range of experimental configurations has been explored to both measure the lifetime of UCN in our magnetic trap and to understand the observed shift in the measured trap lifetime due to systematic effects. The origin of the systematic shift is not completely understood, but as will be shown below, it appears to arise from a combination of marginally trapped neutrons and material bottling. Lifetime measurements under different experimental conditions yield values for the lifetime in the range of 600 s to 900 s, a spread larger than the statistical uncertainty.

Data was taken using natural helium (with a fractional ^3^He content of approximately 10^−7^) to help understand systematic effects and is shown as the lower curve in Fig. 1. The lifetime of UCN in the trap in the “^3^He” configuration is less than one second. Fits to this data set are consistent with zero or no trapped neutrons. There is also no evidence of neutron activation or neutron-induced luminescence in the data.

Low temperature (< 300 mK) trapping data has been collected over a number of different experimental conditions chosen to probe potential origins of the systematic shift. These conditions include artificially increasing the fluence of neutrons (while keeping the 0.89 nm fluence fixed), varying the length of time we observe neutron decays, ramping the magnetic field to remove marginally trapped neutrons, and numerous tests of the electronics and data acquisition systems (DAQ). In brief, all of the non-magnet-ramping runs yield results that are consistent with one another. The magnet ramping data gives a considerably longer lifetime, $\tau =(833_{-63}^{+74})\;s$ (see Fig. 1), consistent with the known neutron beta-decay lifetime. Thus we believe marginal trapping (probably in combination with the interaction between the walls and the marginally trapped neutrons) to be the primary source of our systematic shift in the data.

We expect that relatively few marginally trapped neutrons will be present in the trap and one can remove these trapped neutrons by lowering the magnetic field to 0.3 of its original value and then raising it again to the original value [3]. This process takes approximately 200 s and is performed immediately after the 0.89 nm neutron beam is turned off. The lowering of the field sweeps out practically all marginally trapped neutrons, while throwing away about 50 % of the “good” (truly trapped) neutrons.

Our hypothesis for the unusually large number and long lifetime for these marginally trapped neutrons is material bottling of these essentially untrapped UCN within the solid walls of the trap cell. Estimates of the UCN potential of the hydrogenous surfaces surrounding the trapping region (tetraphenyl butadiene, GoreTex^1^, etc.) give values of approximately 50 neV. Although the absorption probability per wall interaction is high, we expect that these marginally trapped neutrons (with energies just above the trap depth and likely incident upon the walls at glancing angles) will undergo considerably fewer interactions with the walls than in a simple ballistic model. In fact, these neutrons may interact with the walls only a few times per second, thus having a long lifetime in the cell. We are currently implementing a series of experiments and numerical simulations to further investigate this hypothesis. In any case, if this is the source of the shift, ramping should eliminate it.

### 3.1 Other Investigations

**Fig. 2:**
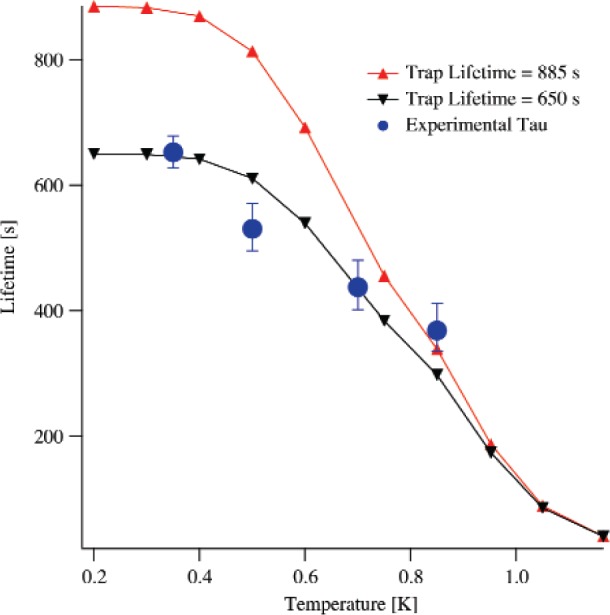
The temperature dependence of the lifetime of UCN in the magnetic trap. The dependence should scale as *T*^7^. Curves are shown for the theoretically predicted dependence for both the world average neutron lifetime and a shorter lifetime (650 s) that closer fits the experimental data.

Data has also been taken at different temperatures in an attempt to verify the theoretical *T*^7^ dependence of the phonon upscattering and, perhaps, aide in the diagnosis of the observed lifetime shifts. Non-ramping data are taken at *T* = 500 mK, *T* = 700 mK and *T* = 850 mK. The extracted lifetimes are shown in Fig. 2. There are a couple of things to note. First, as the temperature is raised, the lifetime in the trap gets shorter as expected. Second, the shape of the temperature dependence appears to track the *T*^7^ dependence if a somewhat shorter intrinsic trap lifetime is assumed. This data is consistent with marginally trapped neutrons causing the shorter lifetime at low temperatures.

Background counts arising from external (to our apparatus) sources continue to be a difficulty; as a point-of-reference, the background count rate drops a factor of five when the reactor is turned off. We significantly reduced background count rates with the addition of an 0.89 nm monochromator and a lead “house” surrounding the apparatus. Nevertheless, the changing experimental conditions from neighboring instruments cause substantial time variation in the backgrounds. We have investigated adding additional shielding of high-density polyethylene, Li-loaded polyethylene, and/or doubling the existing 10 cm of lead. We find that the additional thickness of lead does not significantly change the backgrounds, whereas the addition of 5 cm of both high-density polyethylene and 5 cm of Li-loaded plastic (10 cm total) reduces the background count rates by approximately 20 % to 30 %. Thus we believe that a large fraction of the current backgrounds arises from fast neutron interactions that can be further reduced with additional amounts of hydrogenous materials.

We also investigated placing an external gamma-ray detector just outside the dewar next to the photomultiplier tube (PMT)s to both monitor external rates and hopefully use the data from this detector to normalize the trapping data to minimize these systematic shifts in the backgrounds. The gamma-ray data is somewhat correlated (not as much as we would have hoped) and is presently being used to reject data where the background count rate shows significant changes during the course of a run. Work is proceeding on using this technique to help minimize changes in the backgrounds on a run-to-run basis.

The calibration of the detection system is performed using the 113 Sn emission of a 364 keV conversion electron. The source was mounted on a linear track along the central axis of the cell and the detection effciency was measured at various positions in the trapping region. Factoring in both the positional dependence of the effciency and the beta-decay spectrum of the electrons, we obtain a detection effciency of (48 ± 6) % when photomultiplier tube (PMT) threshold levels are set to three or more photoelectrons (see Ref. [2]).

## 4. Future Directions

In order to fully understand the systematic effects that are present in our recent data, an additional improvement to the apparatus is needed to increase the number of trapped neutrons. At present, each lifetime measurement at a given configuration takes approximately 10 days to obtain, so varying the parameters to study the systematics at high accuracy would be both beam-intensive and time-prohibitive. We are proposing to substantially increase the number of neutrons trapped by replacing the present magnetic trap with a significantly deeper trap.

We have on loan from the High Energy Accelerator Research Organization (KEK) in Japan, a high-current superconducting quadrupole magnet that we are in the process of turning into an Ioffe trap. This modification entails adding a superconducting solenoid assembly outside of the existing quadrupole assembly. The trap depth from the new magnet will be a factor of three higher than the present magnet.

Once the KEK trap has been incorporated into a dewar, we plan to take neutron trapping data with the new apparatus at NIST. NIST is presently the best source in the United States for this experiment because of the existence of the 0.89 nm monochromatic beam, the high fluence of neutrons available, and the availability of beamtime. The primary issue in returning to NIST is backgrounds from neighboring instruments.

The increased signal-to-noise resulting from the KEK trap will substantially decrease our susceptibility to backgrounds. Nevertheless, we plan to investigate additional ways to lower our sensitivity to backgrounds. About 50 % of the background events originate in the helium itself and the other half are from high-energy particles scattering in the acrylic in the lightguides.

Once the systematics have been identified and are under control at NIST, we expect to be able to make a measurement of the neutron lifetime with a statistical accuracy of ≈ 3 s in one reactor cycle (39 d). The experiment would then be poised to move to either the new Spallation Neutron Source (SNS) fundamental neutron physics facility or possibly a new external source of UCN. The advantages of these facilities will be the expected lower background rates and the better coupling of the neutron beam into the apparatus. This should allow a measurement with a statistical accuracy of 0.1 s for the same period of running.

## Footnotes

1 Certain commercial equipment, instruments, or materials are identified in this paper to foster understanding. Such identification does not imply recommendation or endorsement by the National Institute of Standards and Technology, nor does it imply that the materials or equipment identified are necessarily the best available for the purpose.
